# Nrf2 Modulates the Hybrid Epithelial/Mesenchymal Phenotype and Notch Signaling During Collective Cancer Migration

**DOI:** 10.3389/fmolb.2022.807324

**Published:** 2022-04-08

**Authors:** Samuel A. Vilchez Mercedes, Federico Bocci, Mona Ahmed, Ian Eder, Ninghao Zhu, Herbert Levine, José N. Onuchic, Mohit Kumar Jolly, Pak Kin Wong

**Affiliations:** ^1^ Department of Biomedical Engineering, The Pennsylvania State University, University Park, PA, United States; ^2^ Center for Theoretical Biological Physics, Rice University, Houston, TX, United States; ^3^ Center for Theoretical Biological Physics , Department of Physics and Department of Bioengineering, Northeastern University, Boston, MA, United States; ^4^ Department of Physics and Astronomy, Department of Chemistry and Department of Biosciences, Rice University, Houston, TX, United States; ^5^ Centre for BioSystems Science and Engineering, Indian Institute of Science, Bangalore, India; ^6^ Department of Mechanical Engineering and Department of Surgery, The Pennsylvania State University, University Park, PA, United States

**Keywords:** epithelial-mesenchymal transition, invasion, jagged1, DLL4, leader cell

## Abstract

Hybrid epithelial/mesenchymal cells (E/M) are key players in aggressive cancer metastasis. It remains a challenge to understand how these cell states, which are mostly non-existent in healthy tissue, become stable phenotypes participating in collective cancer migration. The transcription factor Nrf2, which is associated with tumor progression and resistance to therapy, appears to be central to this process. Here, using a combination of immunocytochemistry, single cell biosensors, and computational modeling, we show that Nrf2 functions as a phenotypic stability factor for hybrid E/M cells by inhibiting a complete epithelial-mesenchymal transition (EMT) during collective cancer migration. We also demonstrate that Nrf2 and EMT signaling are spatially coordinated near the leading edge. In particular, computational analysis of an Nrf2-EMT-Notch network and experimental modulation of Nrf2 by pharmacological treatment or CRISPR/Cas9 gene editing reveal that Nrf2 stabilizes a hybrid E/M phenotype which is maximally observed in the interior region immediately behind the leading edge. We further demonstrate that the Nrf2-EMT-Notch network enhances Dll4 and Jagged1 expression at the leading edge, which correlates with the formation of leader cells and protruding tips. Altogether, our results provide direct evidence that Nrf2 acts as a phenotypic stability factor in restricting complete EMT and plays an important role in coordinating collective cancer migration.

## Introduction

A most devastating feature of cancer is its ability to migrate and invade adjacent tissues (Hanahan and Weinberg, 2011)**.** During invasion by carcinomas, cancer cells can undergo an epithelial-mesenchymal transition (EMT) to gain mesenchymal traits, such as increased motility and invasiveness (Brabletz et al., 2018). Emerging evidence reveals that EMT is not an irreversible, binary process; in contrast, EMT is a reversible transition process with one or multiple hybrid, or partial, epithelial/mesenchymal (E/M) states which can help coordinate the collective invasion of cancer cells (Nieto et al., 2016; Brabletz et al., 2018). These intermediate states arise due to the complex dynamics of cell fate circuits encompassing mutually inhibiting microRNA and EMT transcription factors (Lu et al., 2013; Zhang et al., 2014). Hybrid E/M phenotypes have been associated with more stem cell-like traits, which include resistance to treatment and enhanced aggressiveness in comparison with purely mesenchymal or epithelial phenotypes (Kroger et al., 2019; Pasani et al., 2020). The clinical significance of the hybrid E/M phenotype in collective cancer invasion is evidenced by analyses of circulating tumor cell clusters exhibiting both mesenchymal and epithelial phenotypes (Yu et al., 2013; Liao and Yang, 2020). Furthermore, the hybrid E/M phenotype has been associated with increased metastatic potential and poor clinical outcomes (Papadaki et al., 2019; Quan et al., 2020).

EMT is a complex process involving various signaling pathways (Nieto et al., 2016; Brabletz et al., 2018). Recent mathematical modeling and experimental analyses have demonstrated that a set of phenotypic stability factors (PSFs) can promote and stabilize hybrid E/M state(s) (Bocci et al., 2017; Biswas et al., 2019). For instance, the transcription factor nuclear factor erythroid 2-related factor 2 (NFE2L2), commonly referred to as Nrf2, is implicated in EMT regulation (Riahi et al., 2014) and is associated with poor clinical outcomes in cancer patients (Rojo de la Vega et al., 2018; Taguchi and Yamamoto, 2020). By integrating computational and experimental approaches, we have previously shown that Nrf2 downregulation destabilizes the hybrid E/M state and prevents collective migration in multiple cancer cell lines, while Nrf2 expression stabilizes a hybrid E/M phenotype that co-expresses epithelial and mesenchymal markers (Bocci et al., 2019b).

Notch signaling has also been separately implicated in the regulation of EMT (Brabletz et al., 2011; Bocci et al., 2017; Deshmukh et al., 2021) and the formation of leader cells during collective cancer invasion (Vilchez Mercedes et al., 2021). Notch is an evolutionarily conserved mechanism, which regulates cell-fate differentiation and cell-cell coordination (Henrique and Schweisguth, 2019). When activated in cancer cells, members of the Notch family, such as Notch1 and its ligands Dll4 and Jagged1, are linked to proliferation, survival, and progression (Meurette and Mehlen, 2018). For instance, Dll4 mRNA is upregulated in leader cells, while Notch1 is upregulated in follower cells (Riahi et al., 2015; Dean et al., 2016; Konen et al., 2017; Torab et al., 2020; Torab et al., 2021; Wang et al., 2021). Moreover, despite Dll4 and Jagged1 having opposite functions in the regulation of angiogenesis (Benedito et al., 2009), Jagged1 promotes MYO10 driven filopodial persistence and invadopodium formation in leader cells (Summerbell et al., 2020). Computational models of Notch1 and Jagged1 have predicted the formation of collectively migrating clusters with hybrid E/M phenotypes (Boareto et al., 2016). Finally, Notch signaling is directly coupled to Nrf2 expression *via* reciprocal positive feedback (Wakabayashi et al., 2015; Sparaneo et al., 2016). The complex interplays between Nrf2, EMT, and Notch1 and their roles in collective cancer migration remain poorly understood.

In this study, we investigate the influence of Nrf2 on collective migration of cancer cells, using a combined experimental-computational approach. We experimentally characterized Nrf2 and the EMT related markers, E-cadherin and ZEB1, during collective migration of cancer cells with immunocytochemistry, fluorescence *in situ* hybridization (FISH), and a double-stranded single cell biosensor (Riahi et al., 2013). A 2D scratch assay, which was shown to induce EMT (Gilles et al., 1999; Riahi et al., 2014), and a TGFβ1-enhanced 3D microtumor invasion model were applied. The expression of Nrf2 was modulated by sulforaphane (SFN), Ailanthone (Aila), or by CRISPR-Cas9 gene editing. The influence of Nrf2 on EMT and Notch1 was also investigated by a computational model of the Nrf2-EMT-Notch1 circuit. Specifically, we coupled the intracellular Nrf2-EMT circuitry with the Notch cell-cell communication pathway, which consists of the Notch transmembrane receptor, the Notch intracellular domain (NICD), and the Notch ligands Dll4 and Jagged1. Computations were carried out in a multicellular lattice model that captures the main geometrical features of scratch-induced collective cell migration. Members of the Notch family, including Notch1, Jagged1, and Dll4, were measured at the protein and/or mRNA level in single cells. Lastly, we measured phenotypic behaviors, including the formation of leader cells, the morphology of the leading edge, and the migration speed, in relationship to Nrf2 modulation. The results reveal the important role of Nrf2 in coordinating the hybrid E/M phenotype during collective cell migration.

## Materials and Methods

### Cell Culture and Reagents

RT4 and UM-UC-1 cells (labeled as control) were purchased from American Type Culture Collection (ATCC, United States). The CRISPR-Cas9 knockout cell pools, RT4-Nrf2-KO (labeled as KO) and UM-UC-1-Nrf2-KO, were obtained from Synthego, CA, United States. The epithelial adenocarcinoma cell line, HeLa, was obtained from Abcam (ab255928). DL-sulforaphane (cat. #s4441, Sigma Aldrich, United States) was dissolved in DMSO (cat. #D8418, Sigma Aldrich, United States) according to manufacturer’s instructions. DL-sulforaphane was added to the RT4 cells (labeled as SFN) at a final concentration of 7.5 μM immediately after the scratch assay. RT4 and UM-UC-1 cells were cultured in McCoy’s 5A medium containing 10% FBS and 0.1% Gentamicin (Fisher Scientific, Hampton, NH, United States). HeLa cells were cultured in DMEM containing 10% FBS and 0.1% Gentamicin. All cells were maintained at 37°C in 5% CO_2_, and media were refreshed every 2 days. The following gRNA targeting exon 2 was used for NFE2L2-KO: AUU​UGA​UUG​ACA​UAC​UUU​GG. Knockout cells showed a predicted functional knockout of 63% which was confirmed by Synthego through RT-qPCR showing 75% editing efficiency post expansion at passage 4. All experiments were done between passages 5–8 for the CRISPR/Cas9 Nrf2-KO pool cells in order to maximize the population of knockout cells within the pool. All experiments were performed in polystyrene 24-well plates (cat. # 07-200-740, Fisher Scientific, Hampton, NH, United States).

Double-stranded locked nucleic acid (dsLNA) biosensors and synthetic targets for calibration were synthesized by Integrated DNA Technologies (San Diego, CA, United States). The sequences are available in Supplementary Table S1. The following reagents were used to perform FISH: Stellaris RNA FISH Hybridization Buffer (cat. #SMF-HB1-10), Stellaris RNA FISH Wash Buffer A (cat. #SMF-WA1-60), and Stellaris RNA FISH Wash Buffer B (cat. #SMF-WB1-20). All FISH reagents were acquired from Biosearch Technologies. Transfection reagents for the dsLNA biosensors were acquired from Thermofisher scientific.

### Immunocytochemistry

Cells were washed with warmed 1× Phosphate-Buffered Saline (PBS) twice, followed by fixation with chilled 4% paraformaldehyde (Sigma) in PBS for 15 min. All reagents were kept cold past this point, and incubation was performed at room temperature. Cell permeabilization was performed with 1% Triton X-100 in PBS for 10 min followed by a blocking step with 3% bovine serum albumin (BSA) in PBS for 30 min. The cells were incubated overnight at 4°C with the primary antibodies and then incubated in the dark for 2 h against the secondary antibodies. Primary antibodies used were Nrf2 (1:100, cat. #AF3925; R&D Biosystems), E-cadherin (1:50, cat. #M3612; Agilent Dako), ZEB1 (1:100, cat. #ab124512; Abcam), Jagged1 (1:50, cat. #sc-390177; Santa Cruz Biotechnology), Notch1 (1:100, cat. #ab8925; Abcam), Dll4 (1:100, cat. #PA585931; Thermofisher Scientific). Secondary antibodies used were Alexa-fluor conjugated secondary antibodies (1:1000; Life technologies). Secondary antibody controls (i.e., no primary antibody) were performed to show the labels were specific to the primary antibody. The antibodies were diluted in 3% BSA solution. Wells were washed 3 times with PBS in between each step. Cells were examined using a laser scanning confocal microscope (Leica TCS SP8; Leica Microsystems, Wetzlar, Germany) immediately after the last washing step.

### Cell Migration Assay

The scratch cell migration (“wound healing”) assay was performed to study collective cell migration. Briefly, at 100% confluency, the monolayer was scratched with a sterile 200 μl pipet tip, and the media were refreshed for all wells. Cells were washed with warm 1× PBS before and after scratching. Images were acquired at 0, 24 and 48 h. The experiments were repeated at least three times, and the mean and standard deviation were calculated using ImageJ. Cell migration was expressed as the migration rate in microns per hour (μm/h): (original scratch width – final scratch width)/time.

### Single Cell Gene Expression Analysis and Transfection

The dsLNA biosensors were used to measure mRNA and microRNA (miRNA) expression profiles of target genes in the migrating front of the monolayer (Supplementary Figure S1). The design, characterization, and protocol were described previously (Riahi et al., 2013; Dean et al., 2016). Briefly, the complementary sequence to the target mRNA/miRNA (labeled as probe) is labeled with a fluorophore at the 5′ end. A complementary sequence with a quencher at the 3′ end (labeled as quencher) is designed. All sequences were verified through the NCBI Basic Local Alignment Search Tool for nucleotides (BLASTn). A random probe with no known intracellular targets was also developed as a negative control. For transfection, the probe and quencher were dissolved in 10 mM Tris-EDTA buffer and 0.2 M NaCl before mixing at a 1:2.5 ratio. Then, the probe and Lipofectamine RNAiMAX reagent (cat. #13778075; Thermofisher) were diluted in Opti-MEM media (cat. #31985062; Thermofisher) according to the manufacturer’s protocol. Cells were seeded in a 24-well plate and transfected once they reached 90–95% confluency. Each well contained a total of 1 μg probe with 2 μl Lipofectamine RNAiMAX. The dsLNA biosensors targeting different genes were transfected in separate wells. For the Notch1 siRNA experiment, the knockdown efficiency (61.5%) was characterized by RT-PCR.

### Fluorescence *In Situ* Hybridization

FISH was used to measure mRNA and miRNA expression of target genes in fixed cells in the migrating front. The FISH assay was performed according to manufacturer’s instructions with the probes designed for the single cell biosensors. Briefly, 24 h after the scratch assay, the cells were fixed using 3.7% formaldehyde in 1× PBS and incubated at room temperature for 10 min. Cells were then washed twice with 1× PBS and permeabilized using 70% ethanol in deionized (DI) water for at least 1 h at 4°C. Afterwards, cells were washed with Wash Buffer A (cat. #SMF-WA1-60; Biosearch technologies) for 5 min. Then the miR-200c-3p, Dll4 mRNA and Notch1 mRNA probes were mixed with the hybridization buffer (cat. # SMF-HB1-10; Biosearch technologies) according to the manufacturer’s instructions, covered in foil and placed in the cell incubator at 37°C for 5 h, all subsequent steps were performed in the dark. Then, cells were incubated in Wash Buffer A for 30 min and placed in the incubator. Lastly, cells were incubated with Wash Buffer B (cat. # SMF-WB1-20; Biosearch technologies) for 5 min. Wells were replenished with fresh 1× PBS.

### Imaging and Data Analysis

All images were acquired using a laser scanning confocal microscope (Leica TCS SP8; Leica Microsystems, Wetzlar, Germany). Cell migration images were analyzed in ImageJ and MATLAB.

### Leader-Follower Cell Selection and Quantification

In this study, leader cells in the migrating monolayer are defined as cells at the migrating tip with apparent cell-cell contact with follower cells behind them. To be classified as a leader cell, we considered the distance from the initial boundary, the extent of the migration sprout, or tip, created by the leader cell, and the contact with follower cells. Follower cells were classified as those maintaining direct contact with the leader cell. To quantify number of leader cells per case (i.e., KO, control, and SFN), the number of leader cells was counted per migration edge per case. The value was reported as leaders/mm, that is: (total # of leader cells/1 mm leading edge).

### 3D Microtumor Invasion Assay

Microtumor invasion assays were carried using Cultrex^©^ 3D Spheroid Cell Invasion Assay Kit (cat. #3500-096-K, Trevigen, MD, United States) according to manufacturer’s protocol. Briefly, HeLa cells were stained by incubated with 5 μg/ml CellTracker Green CMFDA Dye in a 35 mm dish at 37°C for 25 min. The cells were then transfected with dsLNA biosensors as previously described. HeLa cells were then incubated in the Spheroid Formation extracellular matrix for 3 days in round bottom low-adherent 96-well plates at a concentration of 5,000 cells per well. The Invasion Matrix, a blend of collagen 1 and basement membrane extract, containing TGFβ1 was added, followed by the addition of media containing SFN or Aila or neither. Each well ultimately contained 10 ng/ml TGFβ1, and 7.5 µM SFN or 0.1 μg/ml Aila or neither. Microtumors were imaged immediately following the addition of the media, then every 24 h for 2 days.

### Statistical Analysis

Data obtained from MATLAB and ImageJ were analyzed using the statistical software GraphPad Prism 9. Experiments measuring mRNA/microRNA levels were performed at least three times in multiple experiments. All other assays were performed at least four times in multiple experiments. In single cell measurement experiments, at least 500 cells per case were analyzed. For first cell layer (i.e., the leading edge) analyses, at least 100 cells per case were measured. The D’Agostino and Pearson test was used to determine data that are not normally distributed. The tests used were: Kruskal-Wallis test with the Dunn’s multiple comparisons test, a Two-Way ANOVA test with a *post-hoc* Tukey test including multiple comparisons across rows and columns, and the ROUT method to identify outliers (Figures 3D, 7B). The four-digit *p*-values are indicated in the figure.

### Multicell Model of the Nrf2-EMT-Notch1 Circuit

We employed a continuous mass action model to describe the biochemical interactions between molecular players in the EMT, Nrf2 and Notch1 circuits. This approach was previously applied to the core regulatory circuits regulating EMT, Nrf2 and Notch1 separately (Boareto et al., 2016; Bocci et al., 2019a; Bocci et al., 2019b). Within a cell, the temporal dynamics for the copy number of any given variable (say, X ) is described by the generic equation:

$$\frac{dX}{dt}=K_{X}-\gamma_{X}X$$ (1)

In Eq. 1, the first term on the right-hand side (RHS) is a production rate with units of number of molecules produced per unit time. The effect of other microRNAs or transcription factors (TF) that induce or inhibit the production of X is modeled by additional functions that modulate the basal production rate. The second term on the RHS models molecule degradation. The full set of equations describing the EMT, Nrf2, Notch circuits and their mutual connections is presented in the Supplementary SA1–SA3.

The effect of transcriptional activation or inhibition exerted by a regulator (say, R) on another given species in the circuit is modeled with a shifted Hill function:

$$H^{S}\left(R,R_{0}\mathrm{,n,\lambda}\right)=\frac{1}{\mathrm{1+}{\left(\frac{R}{R_{0}}\right)}^{n}}\mathrm{+\lambda}\frac{{\left(\frac{R}{R_{0}}\right)}^{n}}{\mathrm{1+}{\left(\frac{R}{R_{0}}\right)}^{n}}\;$$ (2)

Where R is the concentration or copy number of the regulator and $R_{0}$ is the half-maximal concentration parameter expressed in same units of R . Additionally, the Hill coefficient n describes the steepness in transcriptional response with respect to the regulator concentration. Finally, the fold change λ describes the change in target level due to regulation by R ( λ<1 implies that R is an inhibitor, while λ>1 implies that R is an activator). If a species is regulated by multiple TFs, Hill functions are multiplied in the production rate of Eq. 1.

Moreover, microRNAs can inhibit the production of other species in the circuit by binding the target mRNA and facilitating their degradation. This post-translational inhibition is modeled following the microRNA-TF chimera toggle switch model first introduced by Lu and collaborators (Lu et al., 2013). First, a first set of functions $P_{l}\left(\mu ,n\right)$ quantifies the inhibition that a microRNA ( μ ) exerts on the target TF; here, n is the number of sites for microRNA binding to the mRNA. Furthermore, a second set of functions $P_{y}\left(\mu ,n\right)$ describes the corresponding decrease of microRNA due to the degradation of the microRNA/mRNA complex. The explicit form and derivation of these functions is discussed in the Supplementary SA4.

The EMT, Nrf2 and Notch modules are connected as follows. Nrf2 inhibits the production of the mesenchymal transcription factor SNAIL, while being inhibited by both E-cadherin and Keap1. Moreover, Nrf2 is transcriptionally activated by the Notch Intracellular Domain (NICD), and in turn increases the production of Notch, thus effectively forming a double positive feedback loop between the Nrf2 and Notch modules (*see* circuit on Figure 2A).

In the multicellular model, cells are arranged on a two-dimensional hexagonal grid. The intracellular signaling dynamics of Nrf2, EMT and Notch is described within each cell by the coupled system of ODEs. Moreover, the biochemical networks of neighboring cells are coupled *via* ligand-receptor binding between Notch and its ligands, Dll4 and Jagged1. For any given cell ( i ) in the lattice, the numbers of external Notch receptors and ligands available for binding ( $N_{\mathrm{EXT}}^{\left(i\right)}$ , $D_{EXT}^{\left(i\right)}$ , $J_{EXT}^{\left(i\right)}$ ) are calculated as the sums over the cell’s nearest neighbors:

$$N_{EXT}^{\left(i\right)}=\sum_{j\in N\left(i\right)}N_{j}$$ (3a)

$$D_{EXT}^{\left(i\right)}=\sum_{j\in N\left(i\right)}D_{j}$$ (3b)

$$J_{EXT}^{\left(i\right)}=\sum_{j\in N\left(i\right)}J_{j}$$ (3c)

Moreover, to simulate the position-dependent activation of EMT observed in the wound healing experiment, we introduce a gradient of EMT-inducing signal ( T(x,y,t) ) that is secreted at the left end of the lattice (the leading edge), diffuses along the x-coordinate and is removed at the opposite end of the lattice:

$$\frac{dT}{dt}=\frac{\partial^{2}T}{\partial x^{2}}\;$$ (4)

This diffusion dynamics gives rise to a profile where cells close to the leading edge are highly exposed to EMT-inducing signaling while cells in the interior are weakly exposed, thus effectively reproducing how EMT activation depends on distance from the wound in the experimental setup. At the beginning of the simulation (t = 0), the EMT-inducer level is fixed to a constant ( $I_{EXT}$ ) at the leftmost edge of the lattice, which represents the layer’s free end, and is set to zero everywhere else inside the lattice. During the simulation, the EMT-inducer level is maintained at $I_{EXT}$ at the leftmost edge of the lattice to model the constant induction while being kept to zero at the rightmost end to model signal degradation throughout the lattice. Simulation details and complete set of model’s parameters are provided in the Supplementary SA5 and Supplementary Tables S2–S5.

## Results

### Nrf2 Modulates EMT During Collective Cell Migration

**FIGURE 1:**
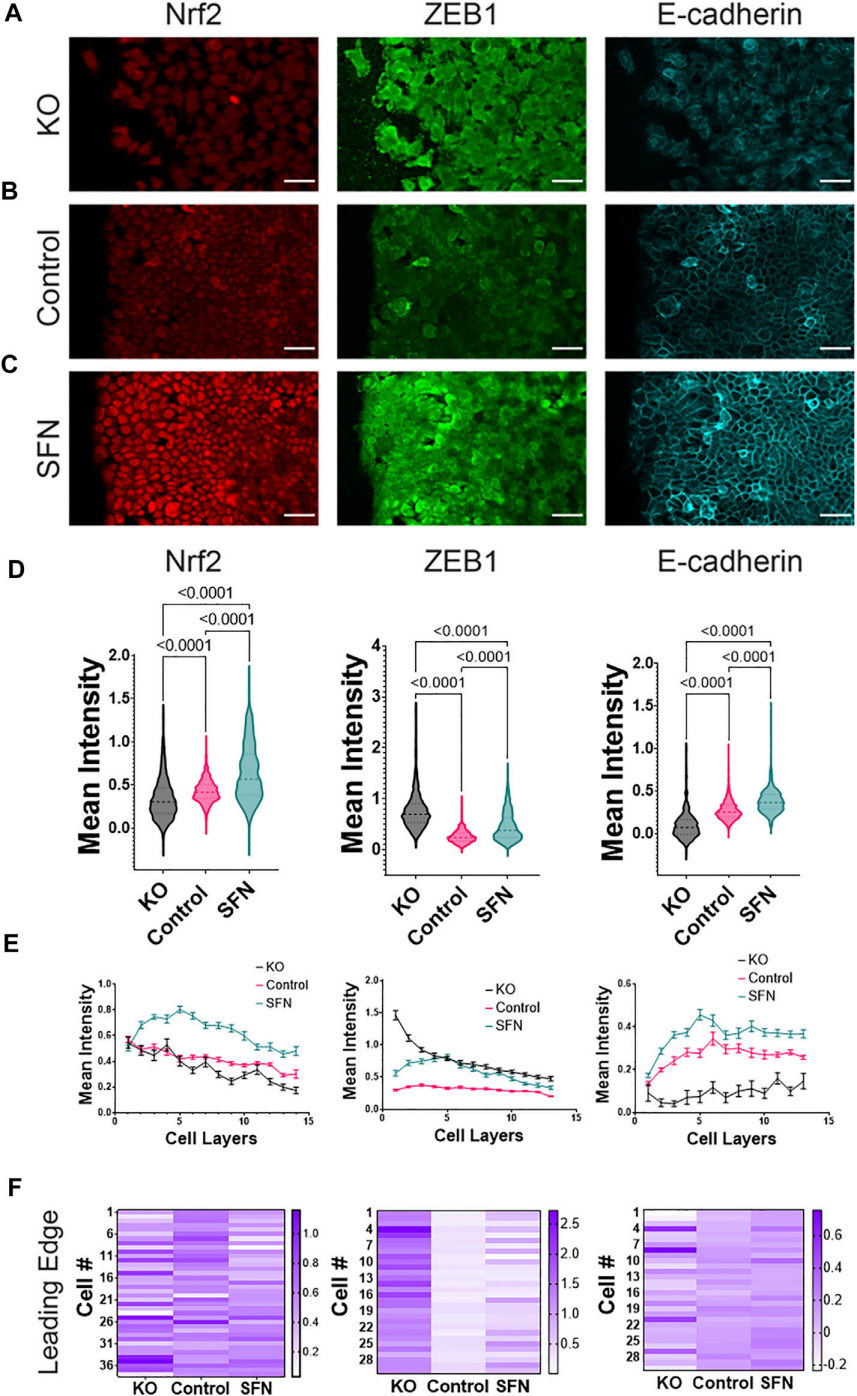
Nrf2 modulates EMT near the leading edge during collective migration. **(A**–**C)** Immunocytochemistry of RT4 bladder cancer cells measuring Nrf2 (left column), ZEB1 (center column), and E-cadherin (right column) protein levels in a cell migration assay for **(A)** CRISPR/Cas9 NFE2L2-KO Pool RT4 cells (KO), **(B)** RT4 cells (Control), and **(C)** sulforaphane treated (7.5 μM, 24 h) RT4 cells (SFN), respectively. Scale bars, 50 μm. **(D**–**F)** Quantification of immunocytochemistry data. **(D)** Average intensity of each cell in the whole monolayer. One-way ANOVA with *post-hoc* Tukey’s test was used to compare across groups (NRF2: KO *n* = 598 cells, Control *n* = 503 cells, and SFN *n* = 564 cells. ZEB1: KO *n* = 385 cells, Control *n* = 1267 cells, and SFN *n* = 1166 cells. E-cadherin: KO *n* = 385 cells, Control *n* = 1268 cells, and SFN *n* = 1166 cells). **(E)** Intensity distribution in the migrating monolayer measured as number of cell layers (position from the leading edge). Data represent mean ± S.E.M. **(F)** Heatmap of representative cells at the leading edge for Nrf2, E-cadherin, and ZEB1, respectively. Each cell at the leading edge is indicated by “Cell #” where cell #1 refers to the first measured cell from top to bottom. Results are from six experiments performed across different days.

We first evaluated the relationship between Nrf2 and EMT during the collective cell migration. Previous investigations in static cell monolayers using RT4 bladder papilloma cells suggest that Nrf2 upregulation enhances the expression of both epithelial and mesenchymal markers (e.g., E-cadherin and ZEB1) while Nrf2 downregulation results in the attenuation of both markers (Bocci et al., 2019b). To study the relationship between EMT and Nrf2 in migrating monolayers, we performed the scratch cell migration assay. Cells were then fixated and fluorescently labeled for Nrf2, E-cadherin, and ZEB1 after 24 h (Figures 1A–C). The resulting images were then segmented into single cells for further analysis. For each gene, data were normalized to control (Figures 1D–F). The complete cell array was analyzed and the mean intensity values were obtained for each cell to obtain an average intensity over the entire migrating monolayer (Figure 1D). Then, data were separated into cell layers (i.e., position relative to the leading edge) to study the spatial distribution in the migrating front (Figure 1E). The intensity distribution at the leading edge itself was further analyzed at the single cell level (Figure 1F).

Nrf2 upregulation *via* sulforaphane (SFN) treatment and Nrf2 downregulation in CRISPR-Cas9 RT4-Pool-knockout (KO) cells resulted in significant enhancement and reduction of Nrf2 across the migrating monolayer, respectively (Figure 1D, left column). Moreover, the Nrf2 distribution showed a significant dependence on the distance from the leading edge. Sulforaphane treatment enhanced the overall level of Nrf2, as expected, but also shifted the maximum level of Nrf2 toward the interior region (layers 3–7) of the migrating cell monolayer (Figure 1E, left column). In contrast, Nrf2 KO resulted in a significant decline in the Nrf2 expression, especially in the interior region of the migrating monolayer. We observed no significant difference across groups at the leading edge (Figure 1F, left column).

We further examined the EMT markers in the migrating front of the monolayer. In the control case, a reduction of E-cadherin was observed near the leading edge, suggesting that cells near the leading edge may undergo EMT, reminiscent of earlier observations (Riahi et al., 2014). For the KO group, we observed an overall reduction of E-cadherin and an enhancement of ZEB1, thus suggesting that cells displayed a more mesenchymal phenotype as compared to control. Both control and Nrf2 KO exhibited EMT activation that gradually fades as a function of distance from the leading edge. In contrast, the sulforaphane group showed high levels of both E-cadherin and ZEB1 compared to the control and thus suggesting a hybrid E/M phenotype (Figure 1D, center and right columns). Furthermore, when examining the spatial distribution, E-cadherin was lowest near the leading edge across all groups, and ZEB1 was highest near the leading edge for the KO group. Interestingly, both E-cadherin and ZEB1 were maximized at rows 3–7 for the sulforaphane group (Figures 1E,F, center and right columns). The formation of hybrid E/M cells was further analyzed by estimating the intensity product of E-cadherin and ZEB1 (Supplementary Figure S2). The intensity product, which signifies cells with both mesenchymal and epithelial signatures, was maximized in the interior region (∼ row 5) of the migrating monolayer for control and sulforaphane. This value was enhanced with sulforaphane treatment, and the peak shifted toward the leading edge. These results suggested that Nrf2 prevents a complete EMT and instead stabilizes a hybrid E/M cell phenotype near but not directly at the leading edge during collective cancer migration.

### *In Silico* Modeling Predicts Nrf2-dependent Increase of the Hybrid E/M Cell Population Near the Leading Edge

**FIGURE 2:**
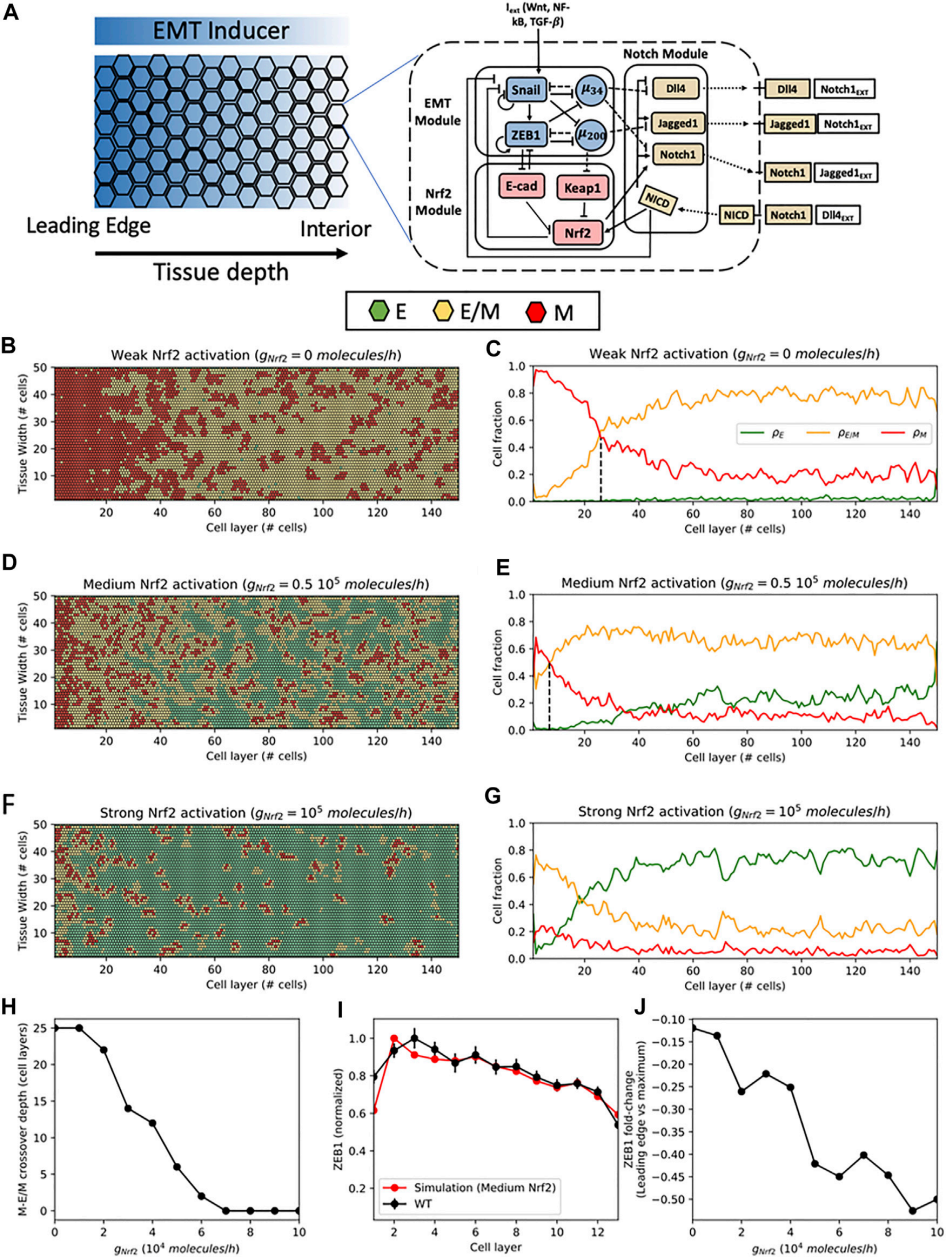
Spatial patterning of cells in the multicell model of cell migration. **(A)** Left: In the multicell model, cells are arranged on a hexagonal lattice. Cells at the leftmost region (leading edge) are highly exposed to an external EMT-inducing signal (indicated by the blue shading), while cells in the interior are weakly exposed. Right: The signaling dynamics within each cell is described by the coupled biochemical network of Nrf2, EMT and Notch. Binding between Notch ligands and receptors of neighboring cells give rise to cell-cell communication. **(B)** Snapshot of the multicell pattern after 120 h of simulation starting from a randomized initial condition for a case of weak Nrf2 activation ( $g_{NRF2}=0$ molecules/hour). Green, yellow and red hexagons depict epithelial (E), hybrid E/M and mesenchymal (M) cells, respectively. **(C)** Fraction of E, E/M, and M cells as a function of distance from the leading edge for weak Nrf2 activation. Black dashed line indicates the M-E/M crossover point. **(D**,**E)** Same as **(B**,**C)** for intermediate Nrf2 activation ( $g_{NRF2}=0.5\times 10^{5}$ molecules/hour). **(F**,**G)** Same as **(B**,**C)** for strong Nrf2 activation ( $g_{NRF2}=10^{5}$ molecules/hour). **(H)** Crossover point where the fraction of hybrid E/M cells becomes larger than the fraction of M cells as a function of Nrf2 production rate. **(I)** Comparison of ZEB1 levels between simulation (red line) and control experiment (black line) in the first 13 cell layers. **(J)** Fold-change in ZEB1 levels between the first and second cell layers as a function of Nrf2 production rate. Result for panels C-E-G-H-I-J are averaged over five independent simulations.

To gain further insight into the role of Nrf2 in regulating EMT, we turned to *in silico* modeling of the underlying regulatory dynamics. We have previously developed circuit models governing EMT-Nrf2 intracellular crosstalk as well as EMT-Notch multicellular signaling dynamics (Boareto et al., 2016; Bocci et al., 2019a; Bocci et al., 2019b). These models predicted that cells could assume up to three different phenotypes: epithelial (E), hybrid E/M, and mesenchymal (M) based on their decreasing levels of the epithelial microRNA miR-200 (Lu et al., 2013) (Supplementary SA5). Nrf2 expression was predicted to be highest in the hybrid E/M phenotype, and Nrf2 induction in E or M cells could potentially induce a transition to the hybrid E/M phenotype. Therefore, Nrf2 was predicted to act as a phenotypic stability factor for the hybrid E/M phenotype. Here, we integrated these models into a more comprehensive framework to investigate how cell-cell and cell-environmental interactions in a collective cell migration scenario modulate the connection between Nrf2 signaling and EMT. In the computational model, the biochemical dynamics within each cell is described by interconnected feedbacks between the Nrf2, EMT, and Notch1 modules. Moreover, binding between Notch1 and its ligands (Dll4 and Jagged1) enables communication between the biochemical circuits of neighboring cells (Figure 2A, right). Simulated cells were exposed to an EMT-inducing signal that diffused throughout the cell layer, thus allowing our model to mimic the spatially-dependent cellular response to the scratch assay. Thus, cells toward the leading edge (the leftmost side) of the lattice are highly exposed to an EMT-inducing signal, while cells in the interior (the rightmost side) of the lattice are only weakly exposed (Figure 2A, left). Varying the EMT inducer level modules the level of EMT. The leading edge can be mostly composed by mesenchymal cells at high EMT induction or by mixed E/M and epithelial cells at low EMT induction (Supplementary Figure S3).

By treating the production rate of Nrf2 as a controllable parameter, we investigated the cell layer’s response to varying levels of Nrf2 induction. Starting from randomized initial conditions, cell populations evolve in time depending on Nrf2 induction and distance from the leading edge. At the basal, or medium, level of Nrf2, the first 5–10 cell layers were mostly composed by mesenchymal cells, while the more interior region was mainly composed by hybrid E/M and epithelial cells (Figure 2D and Supplementary Movie S1). A weaker Nrf2 induction increased the mesenchymal cell population at the migration front while pushing the hybrid E/M cell population to a more interior region of the monolayer (Figures 2B,C and Supplementary Movie S2). Furthermore, the epithelial phenotype was almost completely suppressed. Therefore, a change from medium to weak Nrf2 induction in the computational model recapitulates many of the experimental findings seen when comparing the control with Nrf2 KO cases. In contrast, for a strong Nrf2 induction, the hybrid E/M cell population became dominant even at the leading edge, in good agreement with the high expression of both ZEB1 and E-cadherin in the experimental sulforaphane case (Figures 2F,G and Supplementary Figure S2).

The phenotype distribution can be quantified by a “crossover point,” where the hybrid E/M cell fraction becomes larger than the mesenchymal cell fraction (dashed lines in Figures 2C,E). This transition point depends on several model’s parameters, including the concerted effect of cell-autonomous EMT-induction driven by the signaling gradient and cell-cell communication EMT-induction driven by Notch1 (Supplementary Figure S4). Nrf2 induction, however, moved the crossover point toward the leading edge independently of the other model’s parameters (Figure 2H), thus supporting the role for Nrf2 in preventing complete EMT and stabilizing the hybrid E/M phenotype near the leading edge of the cell monolayer.

The model predicts a drop in the mesenchymal cell fraction at the leading edge, which is instead maximized in the cell layers immediately behind (*see* for instance Figures 2C,E). Invading cells at the free end received Notch1-mediated EMT induction from a smaller number of neighbors, thus resulting in more hybrid E/M cells and less mesenchymal cells. Remarkably, a drop in ZEB1 levels at the leading edge was also observed in both control and SFN experiments (*p* < 0.001), and can be at least semi-quantitatively compared to the simulation results (Figure 2I). Conversely, the KO experiment did not exhibit a ZEB1 drop. It can be speculated that Notch1 plays a lesser role due to the loosen adhesive bonds between the highly mesenchymal cells observed in the KO case, a feature that is not captured by the current model. Moreover, the ZEB1 drop between first and second cell is predicted to increase as a function of Nrf2 (Figure 2J). This trend is qualitatively observed in the experimental model as well, where the SFN case presents a larger drop compared to the control.

Overall, the computational model suggests a role for Nrf2 in modulating the spatial composition of the cell layer by preventing a complete EMT and localizing a population of hybrid E/M cells at the migrating edge, in good agreement with high co-expression of epithelial and mesenchymal markers observed under sulforaphane treatment. The coupled dynamics between Nrf2, EMT and Notch1 drives Nrf2 to act as a brake on EMT, thus increasing the population of hybrid E/M cells in the migrating front.

### Nrf2 Modulates Notch Near the Leading Edge

**FIGURE 3:**
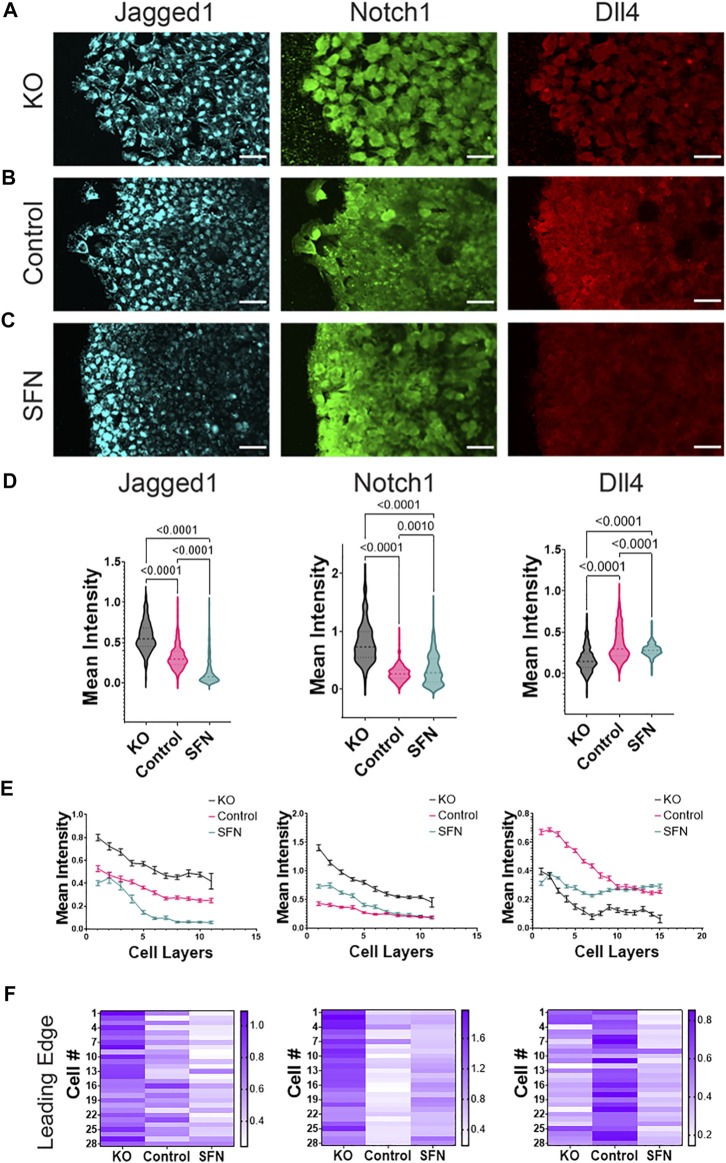
Nrf2 regulates the Notch signaling pathway in the migrating front. **(A**–**C)** Immunocytochemistry of RT4 bladder cancer cells measuring Jagged1 (left column), Notch1 (center column), and Dll4 (right column) protein levels in a cell migration assay for **(A)** CRISPR/Cas9 NFE2L2-KO Pool RT4 cells (KO), **(B)** RT4 cells (Control), and **(C)** sulforaphane treated (7.5 μM, 24 h) RT4 cells (SFN), respectively. Scale bars, 50 μm. **(D**–**F)** Quantification of immunocytochemistry data. **(D)** Average intensity of each cell in the whole monolayer. One-Way ANOVA with *post-hoc* Tukey’s test was used to compare across groups (Jagged1: KO *n* = 307 cells, Control *n* = 526 cells, SFN *n* = 423 cells. Notch1: KO *n* = 307 cells, Control *n* = 370 cells, SFN *n* = 423 cells. Dll4: KO *n* = 460 cells, Control *n* = 1135 cells, SFN *n* = 570 cells). **(E)** Tracing of relative fluorescence intensity per tissue depth measured as number of cell layers. Data represent mean ± S.E.M. **(F)** Heatmap of representative cells at the leading edge for Jagged1, Notch1, and Dll4, respectively. Each cell at the leading edge is indicated by “Cell #” where cell #1 refers to the first measured cell from top to bottom. Results are from four experiments performed across different days.

The spatial patterning determined *via* our computational model depends directly on cell-cell coupling *via* the Notch pathway. From an experimental perspective, Notch has been shown to be a critical component of EMT circuitry (Deshmukh et al., 2021; Simeonov et al., 2021). Also, Notch1 has been shown to regulate collective cell migration (Riahi et al., 2015; Dean et al., 2016; Konen et al., 2017; Torab et al., 2020; Wang et al., 2021). We therefore measured the distributions of Notch components (i.e., Notch1, Dll4, and Jagged1) in the control and under Nrf2 perturbations (Figures 3A–C). In agreement with previous studies (Riahi et al., 2015; Konen et al., 2017; Wang et al., 2021), Notch components were upregulated near the leading edge. Spatial gradients of Notch1, Dll4, and Jagged1 near the leading edge were observed, and the expression levels were dependent on Nrf2. In particular, Jagged1 was negatively correlated with Nrf2 levels, being consistently highest for the KO case and lowest in the sulforaphane case, in the entire monolayer (Figures 3D–F, left column). Notch1 and Dll4 showed mutually exclusive behavior (as expected), but did not respond proportionally to Nrf2 induction. Specifically, Notch1 was lowest for the control case and higher for both KO and sulforaphane, whereas Dll4 was highest for the control case and lowest for KO and sulforaphane cases (Figure 3D, center and right column). This behavior was especially apparent when inspecting cells near the leading edge (e.g., the first five rows), where we noticed a great degree of separation between the control behavior and that of the other two cases (Figure 3E, center and right column). At the leading edge, Notch1 showed lowest intensity values in the control case whereas Dll4 showed the highest intensity values for the control case (Figure 3F, center and right column).

**FIGURE 4:**
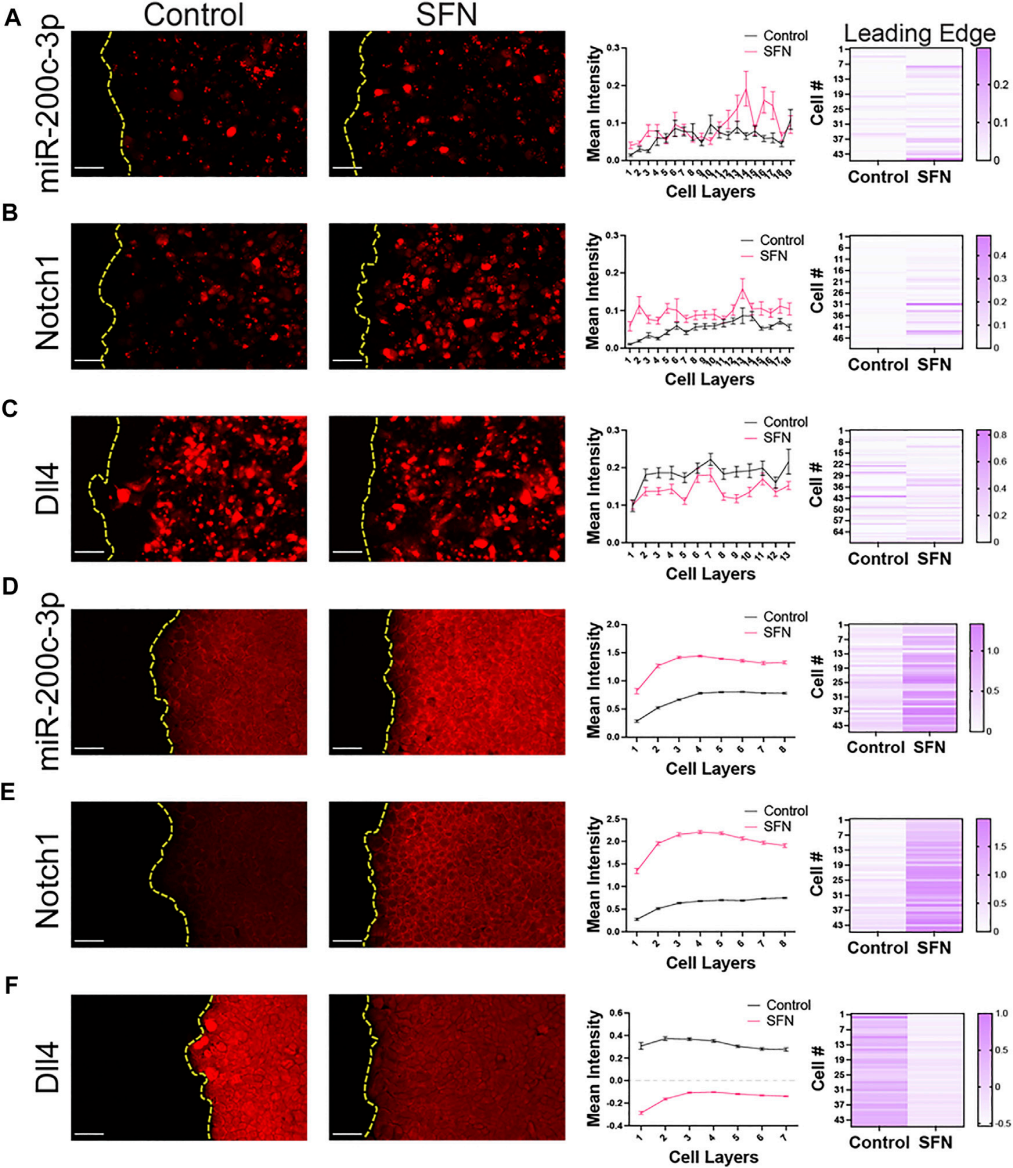
Nrf2 upregulation modulates microRNA miR-200c-3p and Notch1 and Dll4 mRNA in the migrating front. **(A**–**C)** Live single cell gene expression measurements with the dsLNA probes in RT4 cells. From left to right: fluorescence images of control and SFN (7.5 μM, 24 h) cases, tracing of relative fluorescence intensity per tissue depth measured as number of cell layers, and heatmap of representative cells at the leading edge measuring **(A)** microRNA miR-200c-3p, **(B)** Notch1 mRNA, and **(C)** Dll4 mRNA levels in a cell migration assay. **(D**–**F)** FISH assay in fixed RT4 cells. From left to right: fluorescence images of control and SFN cases, tracing of relative fluorescence intensity per tissue depth measured as number of cell layers, and heatmap of representative cells at the leading edge measuring **(D)** microRNA miR-200c-3p, **(E)** Notch1 mRNA, and **(F)** Dll4 mRNA levels in a scratch cell migration assay. Scale bars, 50 μm. Each cell at the leading edge is indicated by “Cell #” where cell #1 refers to the first measured cell from top to bottom. Data represent mean ± S.E.M. The two-way ANOVA with the *post-hoc* Tukey test was performed to compare across groups. At least 500 cells were analyzed for each condition (miRNA-200c-3p, Notch1 mRNA, and Dll4 mRNA). Results are from three experiments performed across different days.

Dll4 mRNA has been reported to be upregulated in leader cells during collective migration (Riahi et al., 2015; Konen et al., 2017; Wang et al., 2021). Particularly, mRNA levels of Dll4 are upregulated in leader cells and exhibit a higher contrast between leader and follower cells when compared to Dll4 protein (Riahi et al., 2015). We therefore directly evaluated the influence of Nrf2 activation on the expression of Notch1 mRNA and Dll4 mRNA. We also measured the miR-200c-3p, which is a key component of the regulatory circuit driving hybrid E/M and can attenuate Jagged1 (Boareto et al., 2016). Specifically, we used a double-stranded single cell biosensor as well as the FISH assay to measure the expression levels of miR-200c-3p, Notch1, and Dll4 in the migrating front (Figure 4). Biosensors were added prior to the cell migration assay to ensure uniform probe internalization (Riahi et al., 2013). Images of live migrating cells were acquired 24 h after scratching to characterize the gene expression. The left panel shows fluorescence images for control and sulforaphane cases measuring miR-200c, Notch1, and Dll4 with the single cell biosensor (Figures 4A–C, left panel) and in the FISH assay (Figures 4D–F, left panel). The right panels indicate the intensity distribution as a function of distance from the leading edge and a representative distribution at the leading edge (Figures 4A–F, right panel).

A gradient of miR-200c-3p was observed in the migrating front. The level of miR-200c-3p was lowest at the leading edge and increased toward the interior region, consistent with the spatial gradient observed in E-cadherin immunostaining. Furthermore, Nrf2 activation by sulforaphane treatment enhanced the level of miR-200c-3p corresponding to an increase in epithelial and hybrid E/M cells. The gradient of miR-200c-3p and the influence of Nrf2 activation were in good agreement with the predictions of the computational model. Furthermore, Nrf2 activation suppressed the average level of Dll4 and enhanced Notch1, similar to the immunocytochemistry analysis. Notably, non-uniform distributions of Dll4 were observed at the leading edge, especially for the control case. In particular, a small number of cells at the leading edge displayed a high level of Dll4. As discussed below, these cells can be identified as leader cells during collective cell migration.

### Computational Modeling Predicts a NRF2-dependent Transition in Notch Signaling Mode and EMT, at the Leading Edge

**FIGURE 5:**
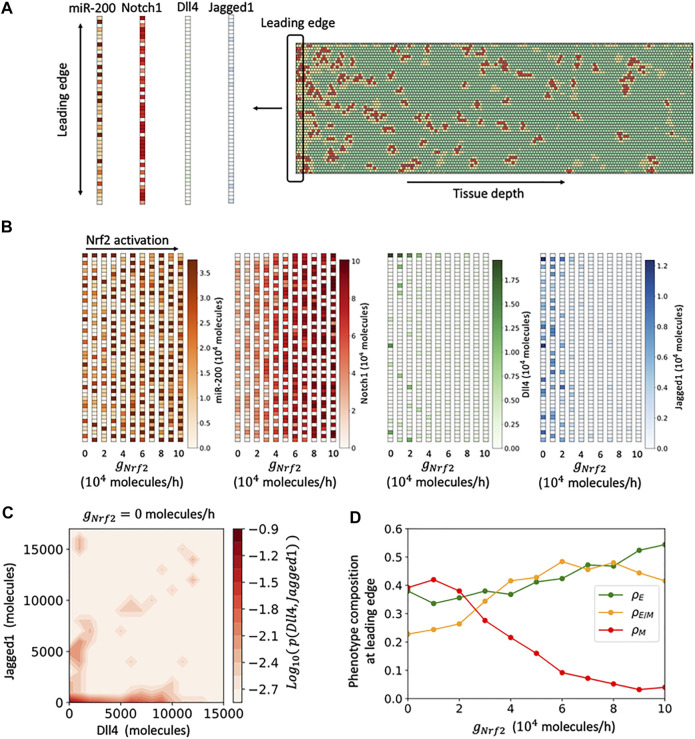
Analysis of the leading edge by the multicell model. **(A)** To conduct leading edge analysis, the expression of miR-200, Notch, Dll4 and Jagged1 is analyzed in the leftmost layer of cells (i.e., the cell layer more exposed to EMT-inducing signal and thus comparable to the experimental leading edge). **(B)** Heatmap of expression levels for miR-200, Notch1, Dll4 and Jagged1 in the leading edge as a function of Nrf2 activation. Each column represents the lattice leading edge for a different level of Nrf2 production rate ( $g_{Nrf2}$ ). **(C)** Log-normalized probability to observe cells with varying levels of Dll4 and Jagged1 in the model’s leading edge under low Nrf2 induction. **(D)** Fraction of Epithelial, hybrid E/M, and Mesenchymal cells in the leading edge as a function of Nrf2 production rate ( $g_{Nrf2}$ ). For panels **(C**,**D)**, results are averaged over five simulations starting from randomized initial conditions.

Next, we returned to the mathematical model to investigate whether the detailed response of Notch1, Dll4 and Jagged1 to Nrf2 modulation could be understood in terms of the interconnected feedbacks between the Notch1 and Nrf2 pathways. Since we were especially interested in Nrf2’s role in mediating collective migration and leader cell formation, we focused our analysis on the leading edge of the multicell model (i.e., the leftmost cell layer that is maximally exposed to EMT-inducing signals) that could be directly compared to the front of migrating monolayer (Figure 5A). As Nrf2 induction increased, the predicted molecular composition of the leading edge changed substantially. Nrf2 induction increased the number of cells with high levels of miR-200 and Notch1 (Figure 5B). The increase of Notch1 can be understood by the mutual positive feedback between NICD, Nrf2, and the Notch receptor (*see* circuit in Figure 2A). Notably, low-Notch cells were still observed for high Nrf2 induction levels due to the negative feedback between Notch1 and Dll4, typically referred to as “lateral inhibition.” Nrf2 induction also decreased the frequency of cells with high Dll4 and high Jagged1 (Figure 5B). In the case of low Nrf2 induction, most cells either expressed high Dll4 or high Jagged1. Interestingly, a small fraction of cells co-expressed both ligands (Boareto et al., 2015). This effect was progressively removed by a stronger Nrf2 induction, as cells expressing either one or both ligands become rarer (Supplementary Figures S5A–C).

In terms of EMT phenotype composition, the leading edge was predominantly composed of mesenchymal cells when Nrf2 induction was low. Conversely, at higher Nrf2 levels, the leading edge was a mixture of hybrid E/M and epithelial cells (Figure 5D). Varying the relative strength of Notch1-Dll4 and Notch1-Jag1 signaling modulates the composition of the leading edge. However, Nrf2 induction restricted the fraction of mesenchymal cells while increasing the fraction of hybrid E/M cells (Supplementary Figures S5D–G). More generally, Nrf2 induction correlated with an average increase in miR-200 and Notch1 expression in the migrating front, as well as decrease of Dll4 and Jagged1 expression, similar to the trend observed in the experiments from control to SFN (Supplementary Figures S5H,I). The trends of Notch1, Dll4 and Jagged1 as a function of Nrf2 induction were confirmed also when inspecting the expression throughout the whole lattice model (Supplementary Figure S6). Noticeably the opposite, experimentally-observed trend of Notch1 and Dll4 from KO to control cannot be reproduced, potentially suggesting that other factors besides Notch-Nrf2 interactions might modulate this response. Overall, the model predicts that Nrf2 induces a transition from a mostly mesenchymal leading edge with strong Dll4 and Jagged1 signaling to a mostly hybrid E/M and epithelial leading edge with high Notch1 expression, in good agreement with the trend observed experimentally when increasing Nrf2 activation from control to sulforaphane.

### Leader Cell Formation Is Optimal for the Control Case and Dll4 Is Highest at the Leading Edge

**FIGURE 6:**
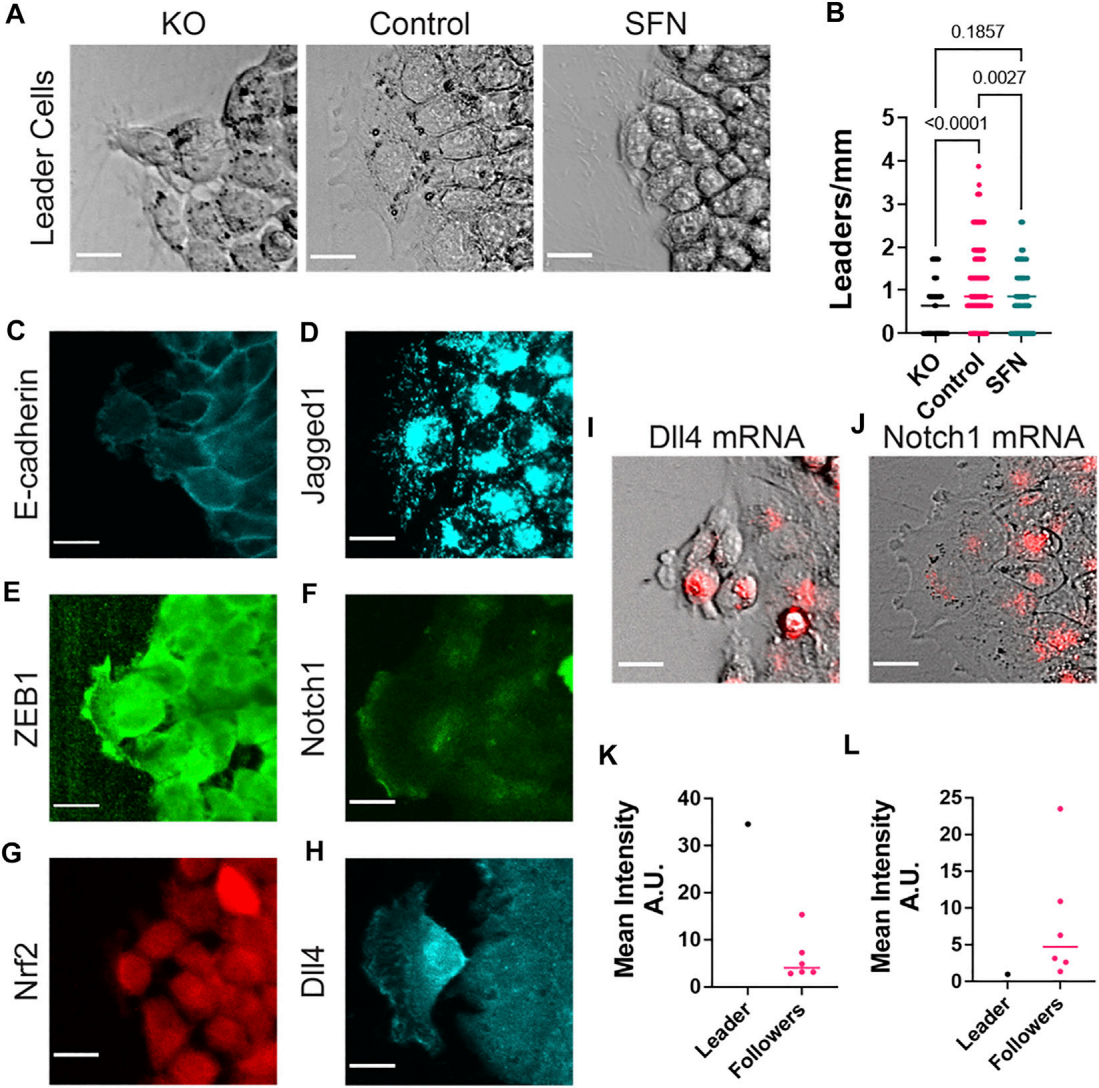
Leader cell formation at the leading edge. **(A)** Representative bright-field images of leader cells after a 24 h cell migration assay for CRISPR/Cas9 NFE2L2-KO Pool RT4 cells (KO), RT4 cells (Control) and sulforaphane treated (7.5 μM, 24 h) RT4 cells (SFN), respectively. **(B)** Plot showing leader cells per millimeter for CRISPR/Cas9 NFE2L2-KO Pool RT4 cells (KO), RT4 cells (Control) and sulforaphane treated RT4 cells (SFN), respectively. One-way ANOVA with *post-hoc* Tukey’s test was used to compare across groups and the ROUT method was used to identify outliers (KO *n* = 46, Control *n* = 100, SNF *n* = 86 leading edges). **(C**–**H)** Representative immunocytochemistry images of leader cells in the control case (RT4 cells) characterizing gene expression for **(C)** E-cadherin, **(D)** Jagged1, **(E)** ZEB1, **(F)** Notch1, **(G)** Nrf2, **(H)** Dll4. **(I**,**J)** Representative overlay images using the dsLNA biosensors to measure mRNA levels of **(I)** Dll4 and **(J)** Notch1, respectively. **(K**,**L)** Mean intensity of leader vs. follower cells for levels of **(K)** Dll4 mRNA (*n* = 6 follower cells) and **(L)** Notch1 mRNA (*n* = 6 follower cells). **(K**,**L)** represent the quantification of one leader cell and its follower cells. Immunocytochemistry data are from four experiments, and dsLNA biosensor data are from three experiments. Scale bars, 20 μm.

The modulation of the Notch ligands Dll4 and Jagged1, which are associated with leader cells (Vilchez Mercedes et al., 2021), suggest Nrf2 may modify the formation of leader cells during collective cancer migration. We thus investigated leader cells at the leading edge (Figure 6). We defined leader cells based on their spatial location at the protruding tips and their interactions with follower cells. Bright-field images at the leading edge revealed distinct morphologies of leader cells for KO, control, and sulforaphane cases (Figure 6A). When treated with sulforaphane, leader cells showed a less mesenchymal phenotype with smaller cell size compared to Nrf2 KO. Leader cells in the control case showed aggressive morphologies, including enlarged cell size and active lamellipodial structures. Moreover, leaders in the control case appeared to entrain a larger number of follower cells when compared to KO and sulforaphane cases (Figure 6A). The control case also exhibited the highest density of leader cells when compared to the other cases (Figure 6B).

We further analyzed molecular markers of leader cells in the control case at the protein and mRNA levels (Figures 6C–H). Leader cells generally showed a low level of E-cadherin and a high level of ZEB1. This is expected as most cells at the leading edge exhibit a mesenchymal phenotype. Leader cells also expressed a low level of Notch1 while upregulating both Jagged1 and Dll4. This observation is particularly interesting as Dll4 and Jagged1 are often assumed as mutually exclusive Notch signaling states (Petrovic et al., 2014; Bocci et al., 2020), and agrees with the model’s prediction that a small population of high Dll4 and high Jagged1 cells exists at the leading edge due to biochemical feedbacks between the Notch, EMT and Nrf2 signaling modules. Furthermore, Jagged1 was relatively uniform in all cells at the leading edge while Dll4 was selectively upregulated in leader cells (Figure 6H). The selective upregulation of Dll4 in leader cells was particularly profound at the mRNA level (Figures 6I,K), which is consistent with previous leader cell investigation, where Dll4 was distinctively upregulated in leader cells at the mRNA level (Riahi et al., 2015). In turn, Notch1 was dramatically downregulated in leader cells while being upregulated in follower cells (Figure 6J,L). This is in agreement with model predictions indicating the mutually exclusive states for Dll4 and Notch1.

### Nrf2 and Collective Cell Migration

**FIGURE 7:**
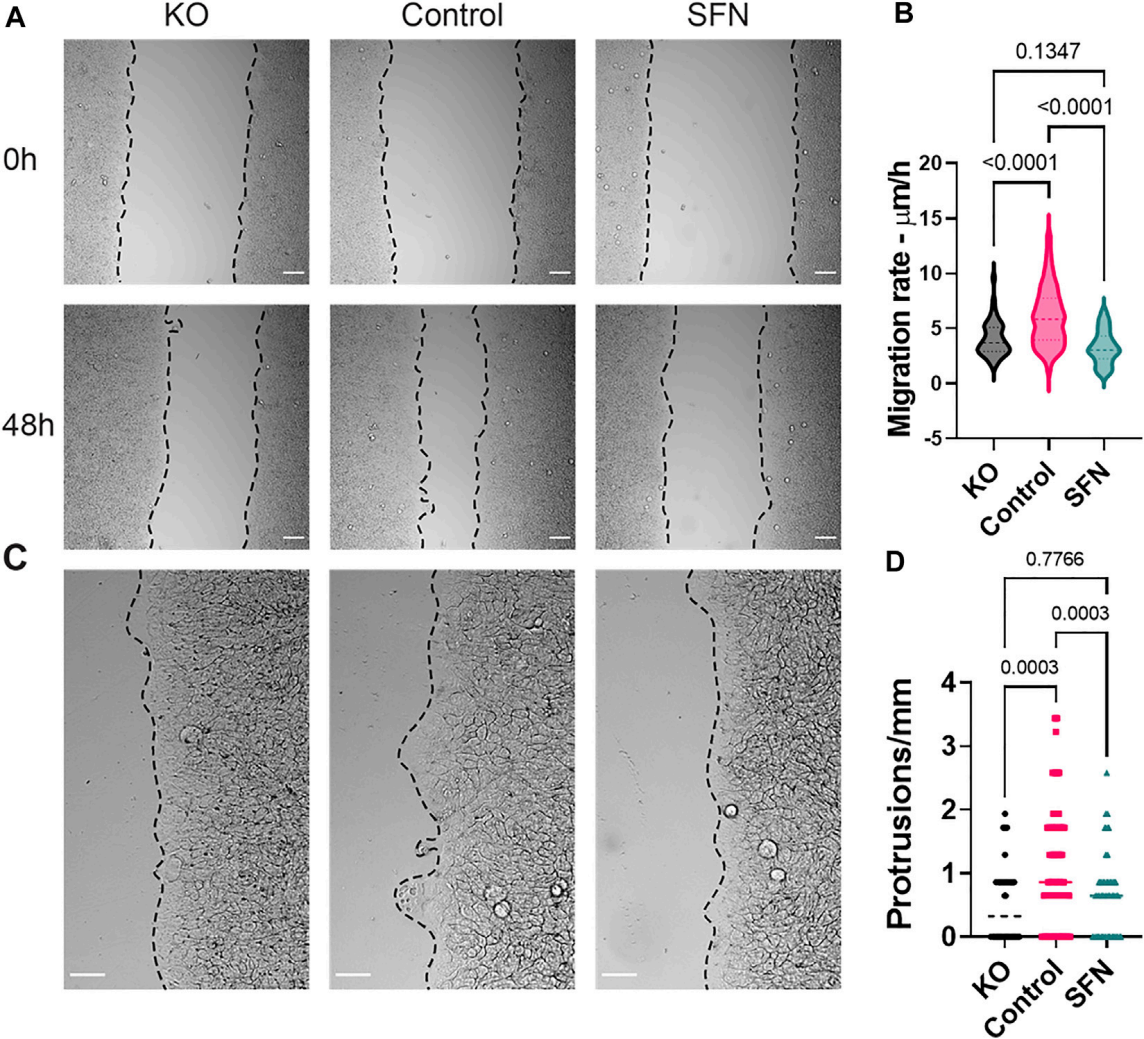
Nrf2 modulations impair collective migration in a 2D model. **(A)** Bright-field images of CRISPR/Cas9 NFE2L2-KO Pool RT4 cells (KO), RT4 cells (Control) and sulforaphane treated (7.5 μM, 24 h) RT4 cells (SFN) for 0 and 48 h migration time points. Scale bars, 100 μm. **(B)** Violin plot of migration rate for KO, control, and SFN, respectively. One-way ANOVA with *post-hoc* Tukey test was used to compare across groups and the ROUT method was used to identify outliers (KO *n* = 44 leading edges, Control *n* = 91 leading edges, SFN *n* = 78 leading edges). **(C)** Representative images illustrating the formation of migration tips. Scale bars, 100 μm. **(D)** Violin plot of protrusions/mm for KO, control, and SFN, respectively. One-way ANOVA with *post-hoc* Tukey test was used to compare across groups and the ROUT method was used to identify outliers (KO *n* = 43 leading edges, Control *n* = 110 leading edges, SFN *n* = 86 leading edges). Results are from four experiments performed across different days.

We analyzed how Nrf2 affects the overall collective migration of cancer cells. The migration of the RT4 monolayer was measured at 0 and 48 h for all cases (Figure 7A). We observed a decrease in migration speed in both the KO and sulforaphane cases (Figure 7B). Specifically, the control case was significantly faster than both the KO and sulforaphane cases (*p* < 0.0001, *n* > 40 cases). This trend correlated with Dll4 expression and the formation of leader cells. Furthermore, “protruding tips” were formed at the leading edge (Figure 7C). The protruding tips often consisted 10–20 cells extended beyond the boundary, resulting in an irregular leading edge. These protruding tips were most profound in the control case (*see* also Figure 6A). The KO and sulforaphane cases, in contrast, displayed relatively uniform boundary and had few or smaller protruding tips (Figure 7D).

### Leader Cells in 3D Microtumor Invasion

**FIGURE 8:**
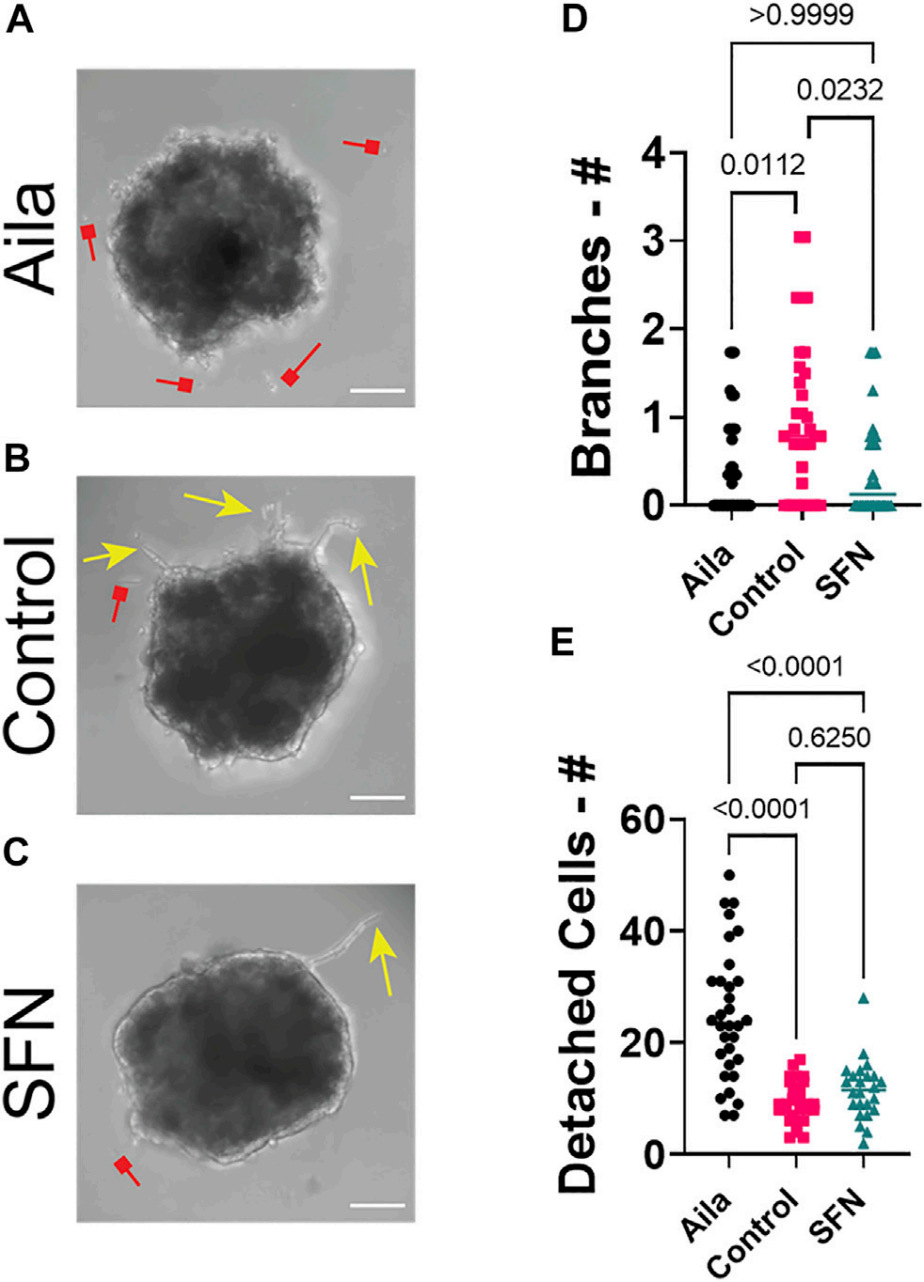
Pharmacological perturbation of 3D invasion. **(A**–**C)** Representative images of 3D microtumors treated with Aila, Control, and SFN, respectively. Yellow arrows represent invasive branches protruding from the spheroids and red arrows represent detached cells. Scale bars, 100 µm. **(D)** Normalized number of invasive branches for Aila, Control and SFN. The D’Agostino and Pearson test was used to determine data that are not normally distributed (Alia *p*-value = 0.0165, Control *p*-value = 0.1437, SFN *p*-value = 0.0372). The nonparametric Kruskal-Wallis test along with the Dunn’s multiple comparisons test were used to compare across groups. **(E)** Average number of detached cells for Aila, Control and SFN, respectively. One-way ANOVA with a *post-hoc* Tukey’s test was used to compare across groups (Aila *n* = 32 spheroids, Control *n* = 28 spheroids, SFN *n* = 24 spheroids). Results are from four experiments performed across different days.

**FIGURE 9:**
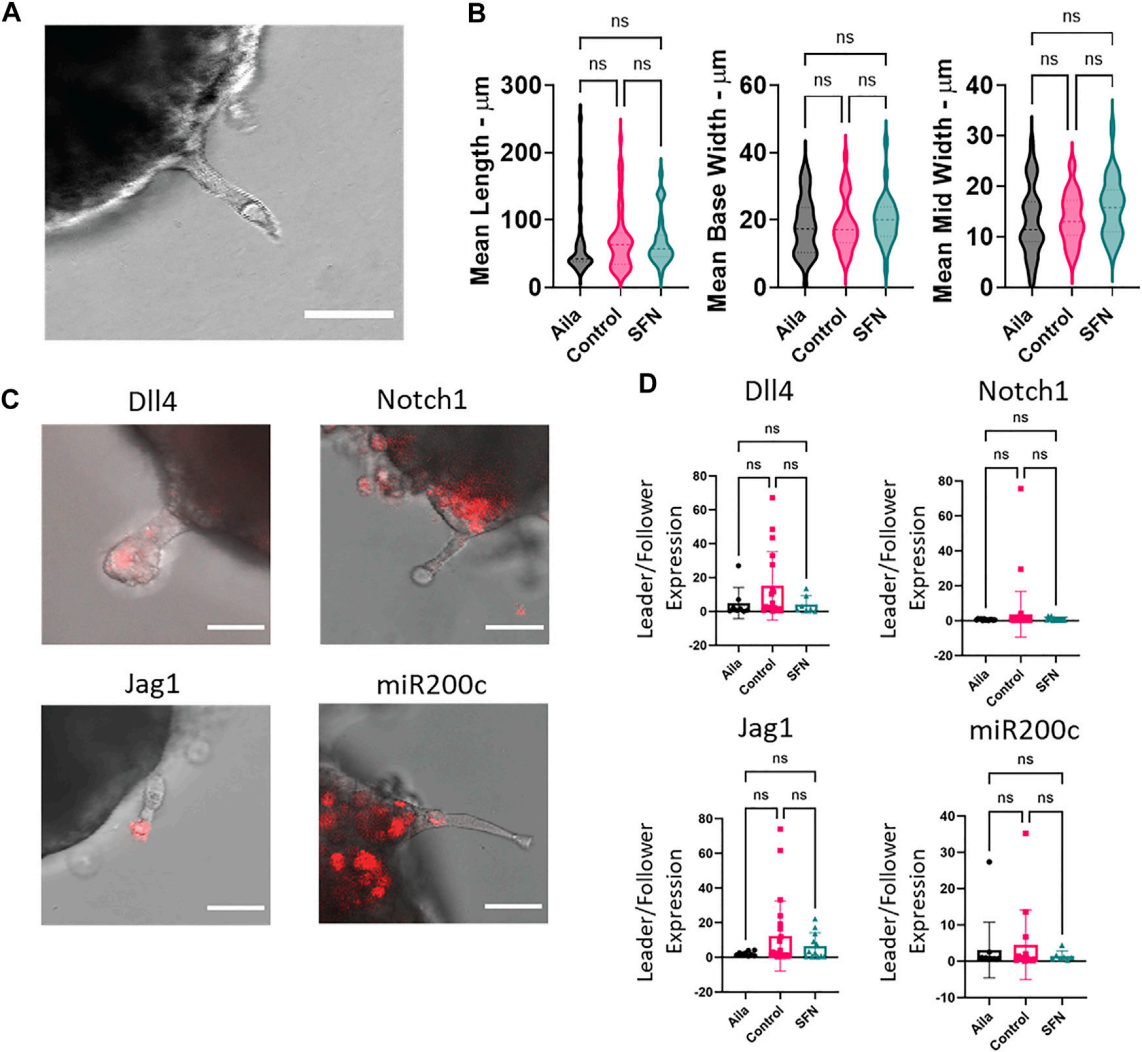
The effects of Ailanthone and sulforaphane on leader and follower cells. **(A)** Representative image of a sprout protruding from a 3D microtumor formed by HeLa cells. **(B)** Unlike the number of sprouts, the morphology (e.g., width and length) of the sprouts was not significantly affected by Alia and SFN treatment. One-way ANOVA with *post-hoc* Tukey’s test was used to compare across groups (Aila *n* = 29 branches, Control *n* = 49 branches, SNF = 27 branches). The experiment was performed three times across different days. **(C)** Overlay images measuring mRNA levels of Dll4, Notch1, Jagged1, and miR-200c in leader and follower cells. Scale bars, 50 µm. **(D)** Relative expressions of Dll4 mRNA, Notch1 mRNA, Jagged1 mRNA, and miR-200c between leader and follower cells in invading sprouts. Leader cells in the control case generally expressed high Dll4 and Jag1 (mean values of leader/follower expression >1) while follower cells expressed higher Notch1 and miR200c across cases (mean values of leader/follower expression <1). The D’Agostino and Pearson test was used to determine data that are not normally distributed (Dll4: Alia *p*-value = 0.4740, Control *p*-value<0.0001, SFN *p*-value = 0.2233. Notch1: Alia *p*-value = 0.2618, Control *p*-value<0.0001, SFN *p*-value = 0.0259. Jag1: Alia *p*-value <0.0001, Control *p*-value = 0.0169, SFN n is too small. mi200c: Alia *p*-value <0.0001, Control *p*-value *p*-value<0.0001, SFN n is too small). For Dll4, Notch1, Jag1, and miR200c respectively: Aila *n* = 9, 13, 8, 12 branches, Control *n* = 22, 37, 18, 14 branches, SNF = 12, 13, 7, seven branches. Results are from three experiments performed across different days.

**FIGURE 10:**
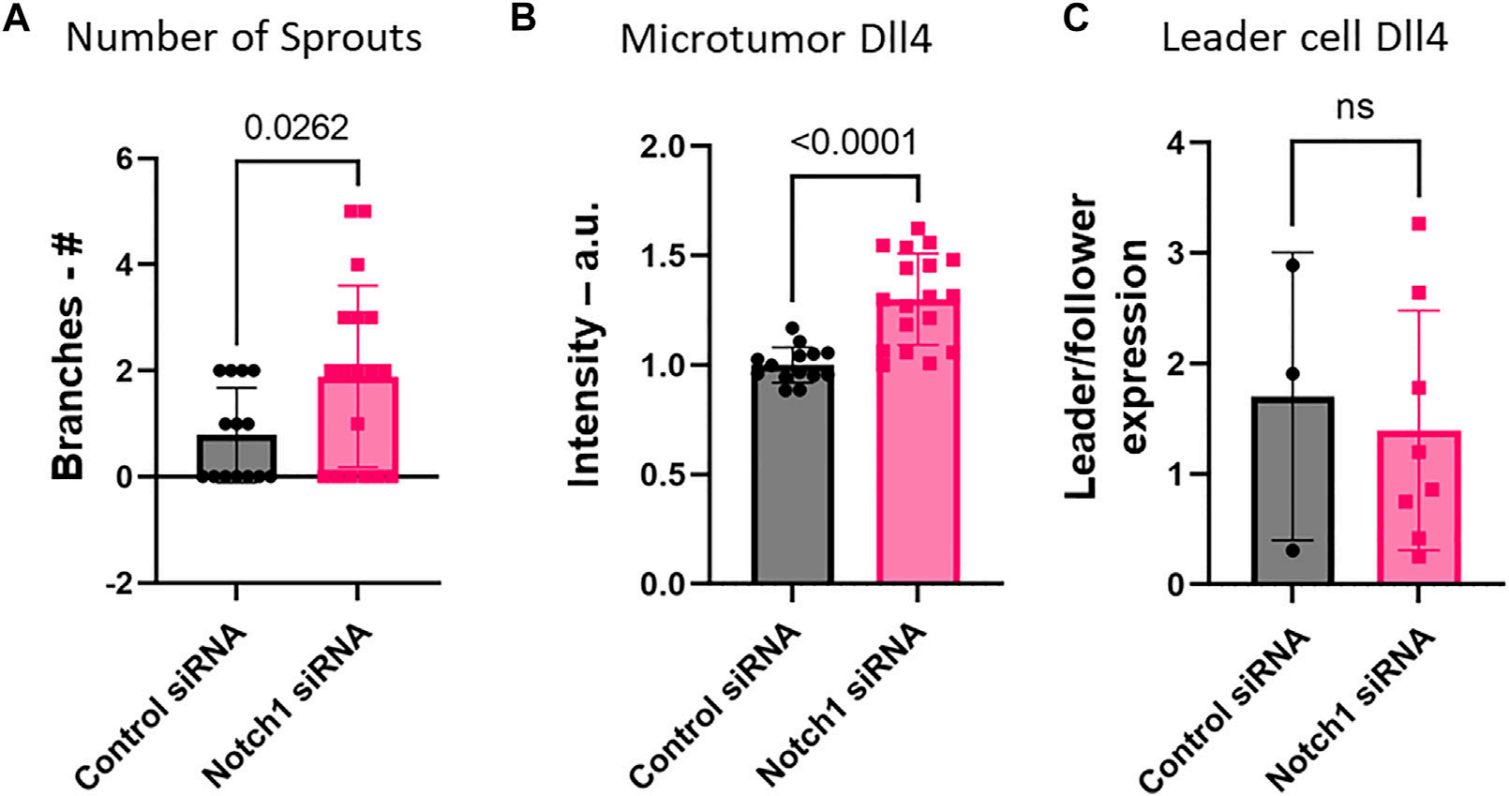
The effect of Notch1 siRNA on TGFβ-induced cancer invasion. **(A)** Notch1 siRNA increased the number of sprouts. The microtumors were formed by HeLa cells. **(B)** Expression of Dll4 mRNA in microtumors was enhanced by Notch1 siRNA compared to control siRNA. **(C)** Once an invading sprout is formed, the expression of Dll4 mRNA in leader cells relative to follower cells was not significantly affected by Notch1 siRNA. Student’s t-test was used to compare the Dll4 expression and the number of branches across groups (ns *p* > 0.05). For **(A**,**B)** control siRNA *n* = 14 spheroids, Dll4 siRNA *n* = 18 spheroids. For **(C)** control siRNA *n* = 3 branches and Dll4 siRNA *n* = 8 branches. Results are from two experiments performed across different days.

We further investigated the formation of leader cells in a TGFβ-induced invasion model (Figures 8–10). In this model, TGFβ1 slightly enhanced the formation of sprouts or branches protruding from the 3D microtumors (Supplementary Figure S7). In additional to SFN, we perturbed the 3D invasion process using Aila, which is known to downregulate Nrf2 (Cucci et al., 2020). The Nrf2 inducer, SFN, reduced the formation of invading sprouts (Figure 8). Similar to what was seen in the Nrf2 KO, application of Aila reduced sprout formation and promoted disassociation of cancer cells from the microtumors (Figure 8). Interestingly, while Aila and SFN modulated the number of sprouts observed, once a sprout was formed, the morphology and distribution of Notch1-Dll4-Jag1 expression were relatively independent of the treatment (Figure 9). Furthermore, transient knockdown of Notch1 by siRNA promoted Dll4 expression, and the number of invading sprouts was enhanced (Figure 10). Similarly, once a sprout is formed, the expression of Dll4 in the leader cells relative to the follower cells was high (leader/follower expression >1) and was independent of the Notch1 knockdown. Overall, we observed an excellent agreement between the 2D and 3D models. These results further support the involvement of Nrf2 and Notch1 in leader cell formation during collective cancer invasion.

## Discussion

This study investigated the role of Nrf2 in modulating the hybrid E/M state(s) of collectively migrating cancer cells. While Nrf2 has been widely studied in the context of antioxidant response and chemoresistance (Lau et al., 2008; Wang et al., 2008), its function in cancer progression and invasion remains poorly understood (Rojo de la Vega et al., 2018; Taguchi and Yamamoto, 2020). Our experimental-computational analysis revealed that the Nrf2-EMT-Notch1 network coordinates cancer cells in the migrating front during collective migration, which represents an important component of the invasion process. In particular, Nrf2 acted as a PSF in suppressing a full EMT and promoting a hybrid E/M phenotype, in a spatially coordinated manner. In the unperturbed condition (i.e., control), the cells in the migrating front displayed a gradient of epithelial to mesenchymal behaviors. The cells near the leading edge were relatively mesenchymal while the cells in the monolayer displayed an epithelial phenotype. It should be noted that even cells at the leading edge maintained cell-cell contact with neighboring cells and expressed a detectable level of E-cadherin, supporting a partial EMT identification instead of a full EMT. In both experimental and computational models, Nrf2 stabilized the hybrid E/M cells, which were positioned at an interior region behind the leading edge. Nrf2 activation was required to maintain the hybrid E/M state of these cells. As indicated in Nrf2 KO, the cells shifted toward a more mesenchymal state, and the level of E-cadherin expression was significantly attenuated in the migrating monolayer. Similarly, Aila disturbed the cell-cell adhesion in the 3D microtumor model and promoted dissemination of individual cancer cells. In contrast, Nrf2 upregulation enhanced the hybrid E/M phenotype and increased both epithelial and mesenchymal markers, especially in the interior region (several rows behind the leading edge) in the migrating monolayer. In addition to RT4, the role of Nrf2 in stabilizing the hybrid E/M state was also observed in another bladder cancer cell line, UM-UC-1 (Supplementary Figure S8). UM-UC-1 Nrf2 KO cells reduced both ZEB1 and E-cadherin in monolayer culture, supporting the notion that Nrf2 functions as a PSF.

Our data implicate a potential function of the Nrf2-EMT-Notch1 network in spatially coordinating collective cell migration. In particular, cancer cells several rows behind the leading edge exhibited upregulated Notch1, Nrf2, and miR-200c while cells at the leading edge expressed ZEB1, Dll4, and Jagged1. The spatial coordination is contributed by the elevated Nrf2-Notch1-NICD activity in the interior region and enhanced ZEB1, which reduced miR-200c (Brabletz et al., 2011) and consequently increased Jagged1, at the leading edge. Importantly, the Nrf2-EMT-Notch1 network promoted the upregulation of Dll4 and Jagged1 at the leading edge in the 2D migrating monolayer and 3D invading sprouts. The expressions of Dll4 and Jag1 appeared to correlate with the formation of leader cells and protrusion tips. For comparison, experiments were performed using HeLa cells in 2D monolayer. The cells expressed observable levels of Notch1, Nrf2 and Nrf2 target genes but a low level of the mesenchymal marker, TWIST1 (Supplementary Figure S9). HeLa cells also expressed relatively low levels of Dll4 and Jag1. The bulk expressions of Dll4 and Jag1 were not significantly affected by the sulforaphane treatment (Supplementary Figures S10, S11). This result supports the idea that the influence of Nrf2 on Notch1-Jag1-Dll4 signaling is associated with EMT, occurring primarily at the leading edge.

The observed spatial coordination of Dll4 and Jag1 may have an important implication for collective cell migration. Dll4 is associated with the formation of leader cells during collective cancer invasion (Riahi et al., 2015; Konen et al., 2017; Wang et al., 2021). Jagged1 is also shown to promote MYO10 driven filopodial persistence for fibronectin micropatterning of leader cells (Summerbell et al., 2020). Our computational analysis shows that the coordination between Dll4 and Jagged1 is highly sensitive to Nrf2 activation at the leading edge. Our predictions indicate that cells expressing both Dll4 and Jagged1 should exist at the leading edge (Figure 5C). Therefore, our results suggest Nrf2 may play a role in the coordination of Dll4 and Jagged1 at the leading edge to regulate different aspects of leader cells. Similar hypotheses have been drawn in contexts of inner ear development and sprouting angiogenesis, where a weak Jagged1 signaling was proposed to further amplify Notch1-Dll4 lateral inhibition by further sequestering Notch1 ligands in high Notch1 receiver cells (Petrovic et al., 2014; Kang et al., 2019). Moreover, Jagged1 is also implicated in inducing partial EMT and cancer stem cell traits and propagating these aggressive traits to neighboring cells *via* Notch1-Jagged1 signaling (Petrovic et al., 2014; Jia et al., 2019; Kang et al., 2019). The precise dynamics acting between Dll4 and Jagged1 and its functional implications in cancer invasion should be further investigated.

Our data indicate that the overall migration speed of collective cancer migration is sensitive to changes in Nrf2 activity. Previous studies report both positive and negative effects of Nrf2 on the collective invasion of various cancer cell types (Pan et al., 2013; Zhang et al., 2019; Wang et al., 2020; Xu et al., 2020; Ko et al., 2021). In general, cancer cell migration can be influenced by multiple factors, such as cell motility, leader cell formation, and proliferation, which can be modulated by Notch and EMT signaling both directly and indirectly. Cancer cells, at least in our model of urothelial bladder cancer, have their maximum migration speed in the unperturbed condition. The migration speed correlated with Dll4 expression and the formation of protruding tips and leader cells. Furthermore, EMT is associated with the motility and proliferation of cancer cells (Nieto et al., 2016; Brabletz et al., 2018). In our model, Nrf2 activation by sulforaphane treatment promoted the hybrid E/M phenotype and enhanced proliferation (Supplementary Figure S12). Additional signaling pathways and molecular programs, such as stemness and metabolic switching (Han et al., 2013; Jolly et al., 2015; Commander et al., 2020), may also be involved in the regulation of the cancer invasion process. All these factors can contribute to the migratory behavior in a context specific manner, and the interrelated roles of Nrf2 on EMT and Notch1 may explain the discrepancy on the functions of Nrf2 on the collective cancer migration.

This study applied an integrated experimental-computation approach to investigate the function of Nrf2 in collective cancer migration. The computation prediction based on our theoretical frameworks generally captured the observed hybrid E/M phenotypes. Other mathematical models have been proposed, which shed light onto the EMT spatiotemporal patterning during cancer invasion and the persistence of the EMT program (Ramis-Conde et al., 2008; Celia-Terrassa et al., 2018; Mukhtar et al., 2021). A recent model integrated Notch signaling and E-cadherin production to investigate changes in adhesion through contact-dependent signaling (Mulberry and Edelstein-Keshet, 2020). The strength of our theoretical framework is the explicit description of cell-cell communication through the Notch pathway and its biochemical feedbacks with EMT and Nrf2. By explicitly coupling intracellular and intercellular biochemical signaling, we provide a predictive framework that generates falsifiable predictions about the role of Notch in regulating EMT and collective cell migration. We note several limitations of the study. For instance, the current model could reproduce well the response of EMT and Notch1 upon Nrf2 upregulation *via* sulforaphane, but could not capture the decrease of Dll4 and increase of Notch1 observed in the Nrf2-KO, potentially pointing to loss of adhesion and weakening of Notch signaling between mesenchymal migrating cells as an important element to integrate into future modeling efforts. The complex interplay between signaling and migratory dynamics also underscores future theoretical challenges that are not explicitly considered in our current model, including *1*) the coupling of biochemical and mechanical regulation of cell migration, *2*) the effect of cell proliferation on cell patterning, and *3*) the context-specificity of the EMT program in terms of both transcriptional response and number of intermediate phenotypes in the EMT spectrum (Mcfaline-Figueroa et al., 2019; Cook and Vanderhyden, 2020; Deng et al., 2021). Furthermore, in order to overcome the limitations of using pharmacological methods such as sulforaphane, additional experimental models involving specific ways of perturbing one or multiple genes (i.e., gene editing techniques such as CRIPSR/Cas9), epistasis studies modulating Notch and the EMT network, and physiologically relevant invasion models should be performed to investigate the impact of the Nrf2-EMT-Notch1 network on cancer invasion. Future experimental and computational investigations will be required to fully understand the role of Nrf2 on collective cancer invasion.

## Data Availability

The original contributions presented in the study are included in the article/Supplementary Material, further inquiries can be directed to the corresponding authors.
